# Using Provocative Discography and Computed Tomography to Select Patients with Refractory Discogenic Low Back Pain for Lumbar Fusion Surgery

**DOI:** 10.7759/cureus.514

**Published:** 2016-02-27

**Authors:** Mengqiao Alan Xi, Henry C Tong, Daniel K Fahim, Mick Perez-Cruet

**Affiliations:** 1 Oakland University William Beaumont School of Medicine; 2 Michigan Head and Spine Institute, Oakland University William Beaumont School of Medicine; 3 Department of Neurosurgery, Oakland University William Beaumont School of Medicine

**Keywords:** discogenic pain, low back pain, computed tomography, discography, annular tear, degenerative disc disease

## Abstract

Background Context

Controversy remains over the use of provocative discography in conjunction with computed tomography (CT) to locate symptomatic intervertebral discs in patients with chronic, low back pain (LBP). The current study explores the relationship between discogenic pain and disc morphology using discography and CT, respectively, and investigates the efficacy of this combined method in identifying surgical candidates for lumbar fusion by evaluating outcomes.

Methods

43 consecutive patients between 2006 and 2013 who presented with refractory low back pain and underwent discography and CT were enrolled in the study. For this study, "refractory LBP" was defined as pain symptoms that persisted or worsened after 6 months of non-operative treatments. Concordant pain was defined as discography-provoked LBP of similar character and location with an intensity of ≥ 8/10. Fusion candidates demonstrated positive-level discography and concordant annular tears on CT at no more than two contiguous levels, and at least one negative control disc with intact annulus. Surgical outcomes were statistically analyzed using Visual Analog Scale (VAS), Oswestry Disability Index (ODI), and Short Form-36 (SF-36) for back-related pain and disability preoperatively, and 2 weeks, 3, 6, 12, and 24 months postoperatively.

Results

Annular tears were found in 87 discs. Concordant pain was reported by 9 (20.9%) patients at L3-L4, 21 (50.0%) at L4-L5, and 34 (82.9%) at L5-S1; pain occurred significantly more often in discs with annular tears than those without (p<0.001). Painless discs were independent of annulus status (p=0.90). 18 (42%) of the original 43 patients underwent lumbar fusion at L3-L4 (n=1(6%)), L4-L5 (n=6 (33%)), L5-S1 (n=5 (28%)), and two-level L4-S1 (n=6 (33%)) via a minimally invasive transforaminal lumbar interbody fusion (MITLIF) approach with the aim to replace the nucleus pulposus with bone graft material. Median follow-up time was 18 months (range: 12–78 months). VAS, ODI, and SF-36 scores demonstrated significant improvements at 10 out of 12 postoperative time points compared with preoperative baseline.

Conclusions

Lumbar discography with post-discography CT can be an effective method to evaluate patients with discogenic back pain refractory to non-operative treatments. Those patients with one- or two-level high concordant pain scores with associated annular tears and negative control disc represent good surgical candidates for lumbar interbody spinal fusion.

## Introduction

Over two-thirds of individuals experience chronic low back pain (LBP) in their lifetime [1]. Some of the most common causes of low back pain include the biochemical degeneration of the intervertebral disc (IVD), spinal stenosis, and disc herniation [1-3]. Posterolateral and interbody fusion techniques are frequently considered for patients with one- or two-level degenerative disc disease whose symptoms are unresponsive to conservative treatment [4]. Nevertheless, pre-operative diagnostic techniques that may identify those patients with degenerative disc disease without significant neurological compression who may benefit from surgical intervention remain elusive.

A number of factors adversely affect the cellular metabolism of the intervertebral disc, including cigarette smoking, atherosclerosis of segmental arteries, and sudden or sustained weight overload [1]. A common finding associated with the onset of disc degeneration is radial annular tears, which can be visualized radiologically with injection of contrast material into the nucleus pulposus followed by computed tomography (CT) imaging. However, these tests have high false-positive rates of approximately 33% - 35% [3,5]. Magnetic resonance imaging (MRI) studies have also shown that anatomical disruption does not always correlate with symptomatic pain [5-7]. This obscures the necessity for surgical correction and leads to overtreatment or undertreatment [8]. Based on these findings, it seems that imaging alone is not sufficient for locating the pathogenic intervertebral disc.

Provocative discography is a useful technique that complements CT by differentiating anatomically abnormal discs that generate pain from those that do not [8-11]. Discography has shown value as a screening tool. Margetic et al., reported in a randomized trial that, with positive radiological evidence, positive discography was associated with clinically significant postoperative improvement, while negative discography was not [12]. Other outcome-based studies have evidenced against the stand-alone use of discography in chronic low back pain due to poor results [13]. Symptomatic patients with negative MRI and negative discography demonstrated similarly unsatisfactory outcomes, but as many as 75% symptomatic patients with positive MRI and positive discography were associated with surgical success [14]. In the present study, positive findings on both discography and imaging were correlated to select patients for lumbar interbody fusion surgery, and postoperative outcomes were investigated.

## Materials and methods

### Patients

43 consecutive patients (23 (53%) men, 20 (47%) women, mean age 51.8±11.2) who presented with refractory low-back pain were included in the study. For this study "refractory" patients were defined as those whose pain symptoms persisted or worsened after a minimum of 6 months of non-operative treatments, including but not limited to analgesics, physical therapy, lifestyle modifications, and steroid injections. Comorbidities are summarized in Table 1.

**Table 1:** Comorbidities

Comorbidities	
Smoking	18 (50%)
Urinary incontinence	10 (24%)
Hypertension	10 (23%)
Breathing problems	8 (19%)
Diabetes	6 (14%)
Cardiovascular disease	4 (9%)

For clinical consistency, discography was performed on all patients by the same physician (H.C.T.). All lumbar fusion surgeries were carried out by the senior author (M.J.P.).

### Discography

The benefits and risks of discography were thoroughly discussed with the patient prior to signing informed consent. Sterile fields were created using standard practices for surgical procedures. Fluoroscopy was used to identify the targeted vertebral interspaces and the bony landmarks of the planned, left-sided posterolateral approach. The skin, subcutaneous tissue, and muscle in line with the planned approach were anesthetized with 1% Lidocaine to the posterior portion of the superior articular process. Under fluoroscopy, a 22G or 23G 6" needle was inserted and directed into the central nucleus of each IVD. In this study, the most commonly examined levels were L3-L4, L4-L5, and L5-S1. Next, the testing phase began with 9cc Omnipaque (Winthrop-Breon Laboratory, NY, NY) mixed with 1cc clindamycin 150mg/cc injected separately into each of the tested IVDs in a random manner. The patient maintained constant communication with the examiner during the test. The patient was not informed of which level was being injected during questions about pain response. Injection volume, pain response, and concordance were recorded. Throughout the procedure, the patient was awake and alert. The patient was able to voluntarily move bilateral legs, feet, and toes upon command following the procedure. All procedures were performed by author H.C.T.

### Criteria for surgery

Concordant pain was defined as provoked, low-back pain of similar character, location, and intensity. For this study, intensity threshold for concordance was set at ≥8/10 on a 10-point scale. Subsequent CT evaluation was performed at each injected level. Radial trans-annular tears that allowed extravasation of radio-opaque dye from the intervertebral disc were considered positive morphological findings. Discs with contained dye within the nucleus pulposus were considered negative. Candidates for interbody arthrodesis satisfied all of the following criteria:

1. Demonstrated concordant pain on discography, with annular tears at the same level(s) on post-discography CT.

2. Pain with annular tears was found at one or two, but not three or more, contiguous intervertebral levels.

3. At least one level of negative control disc (negative discography and negative CT) existed.

**Figure 1:**
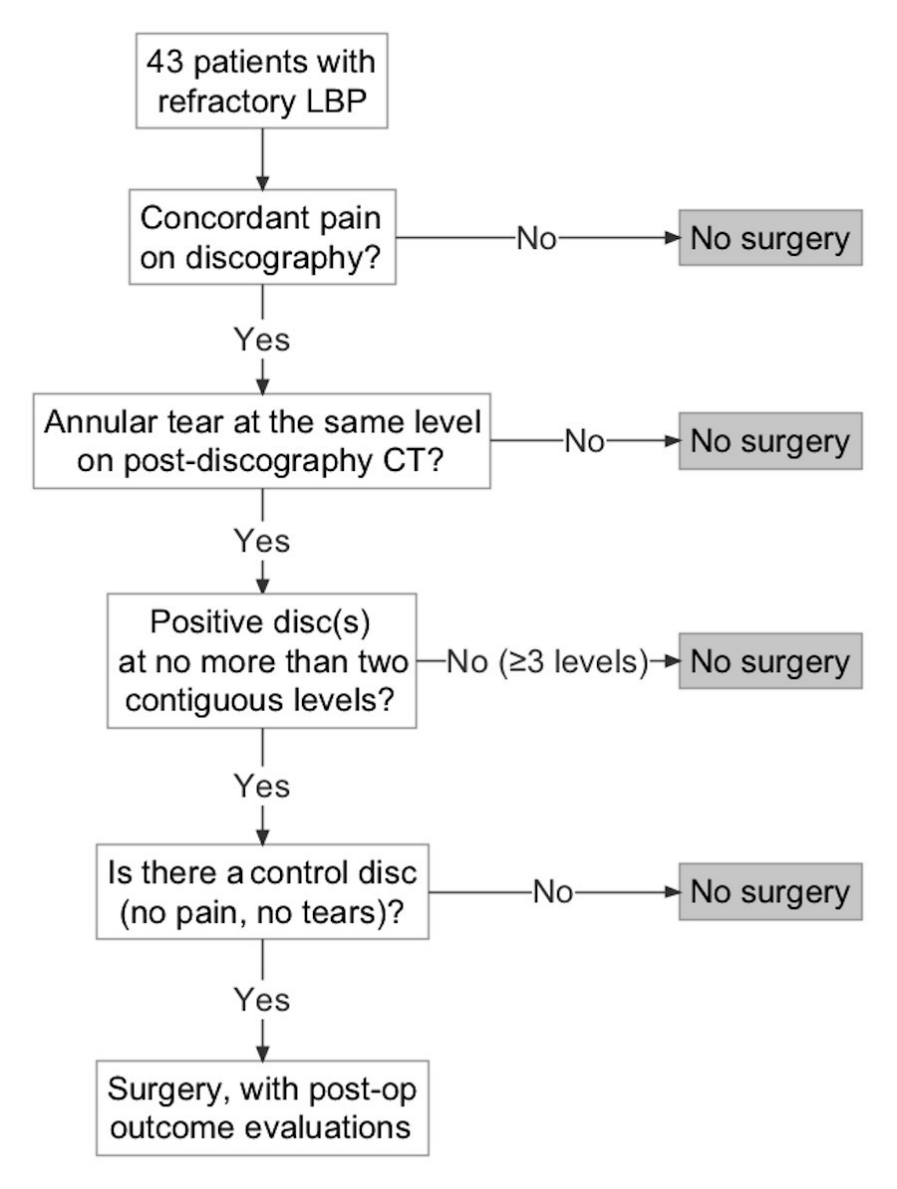
Decision-making algorithm for interbody fusion

The decision-making algorithm is illustrated in Figure 1.

### Surgery and clinical outcome scoring

Lumbar interbody fusion was achieved via a minimally invasive transforaminal approach previously described [15]. Other options such as anterior lumbar interbody fusion (ALIF), posterior lumbar interbody fusion (PLIF), and extreme lateral interbody fusion (XLIF) were deemed inappropriate by the surgeon based on individual patho-anatomy; all options were fully discussed with the patients before informed consent was obtained. In-situ fusion and other approaches were not studied in this series in order to maintain technical homogeneity. Postoperative outcomes were evaluated with visual analog scale (VAS) for low-back pain, Oswestry Disability Index (ODI) for back-related functional disability, and Short Form-36 (SF-36) for physical and mental quality of life. Patients were asked to complete these questionnaires at different time points, namely preoperative, 2 weeks, 3, 6, 12, and 24 months post-operative.

### Statistical analysis

The Student’s t-test was used to comparatively analyze the difference between dye injection volumes in painful discs versus non-painful discs, and to evaluate changes in VAS, ODI, and SF-36 scores from preoperative baseline. The chi-square test was used to evaluate the relationship between annular tears and concordant pain. Statistical significance was defined as p<0.05. All statistical analyses were performed with Microsoft Excel software (version 2010; Microsoft Corporation; Redmond, Washington).

## Results

### Discography

A total of 129 intervertebral levels were examined with 126 discs at risk and the remaining 3 previously fused. Disc levels that exhibited concordant pain were L5-S1 (80.5%), L4-L5 (50.0%), and L3-L4 (20.9%). Annular tears were found in 30 (88.2%) patients who reported concordant pain at L5-S1, 18 (85.7%) at L4-L5, and 6 (60.0%) at L3-L4. These results are summarized in Table 2.

**Table 2:** Pain findings on discography and annular findings on post-discogram CT at L3-4, L4-5, and L5-S1

	L3-4	L4-5	L5-S1
Pain score	4.84 ± 4.10	7.36 ± 4.97	9.30 ± 3.98
Concordant pain	9 (20.9%)	21 (50.0%)	33 (80.5%)
Annular tears	20 (43.9%)	33 (78.6%)	34 (79.1%)
Fusion recommended	6	18	30

The volume of injected dye was significantly higher in patients with concordant pain (1.91±1.19 ml, n=61) than those without (1.58±1.05 ml, n=65) (p<0.05). The volume of injected dye was also significantly higher in patients with annular tears (2.01±1.28 ml, n=87) than those without (1.25±0.54 ml, n=39) (p<0.001).

### Post-discography CT

**Figure 2:**
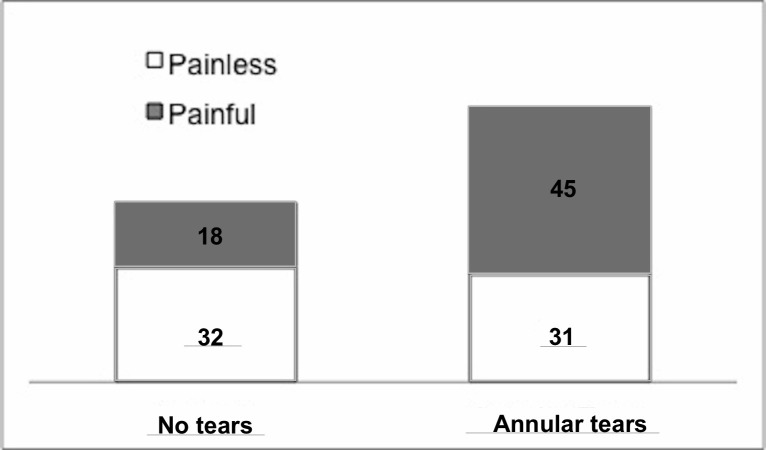
Relationship between discography and imaging results 76 of the 126 discs (60.3%) studied exhibited trans-annular tears. 45 (59.2%) of these discs showed concordant pain on discography, while 31 (40.8%) did not. 50 of the 126 discs (39.6%) studies did not exhibit trans-annular tears. 18 of these discs (36.0%) showed concordant pain on discography, while 32 (64.0%) did not. Discs showing concordant pain on discography occurred significantly more often with annular tears (p<0.001) than without annular tears. Discs with negative discography showed no association with annular status (p=0.90).

Concordant pain occurred significantly more often in discs with annular tears than those without (p<0.001). Painless discs were independent of annulus status (p=0.90) (see Figure 2).

**Figure 3:**
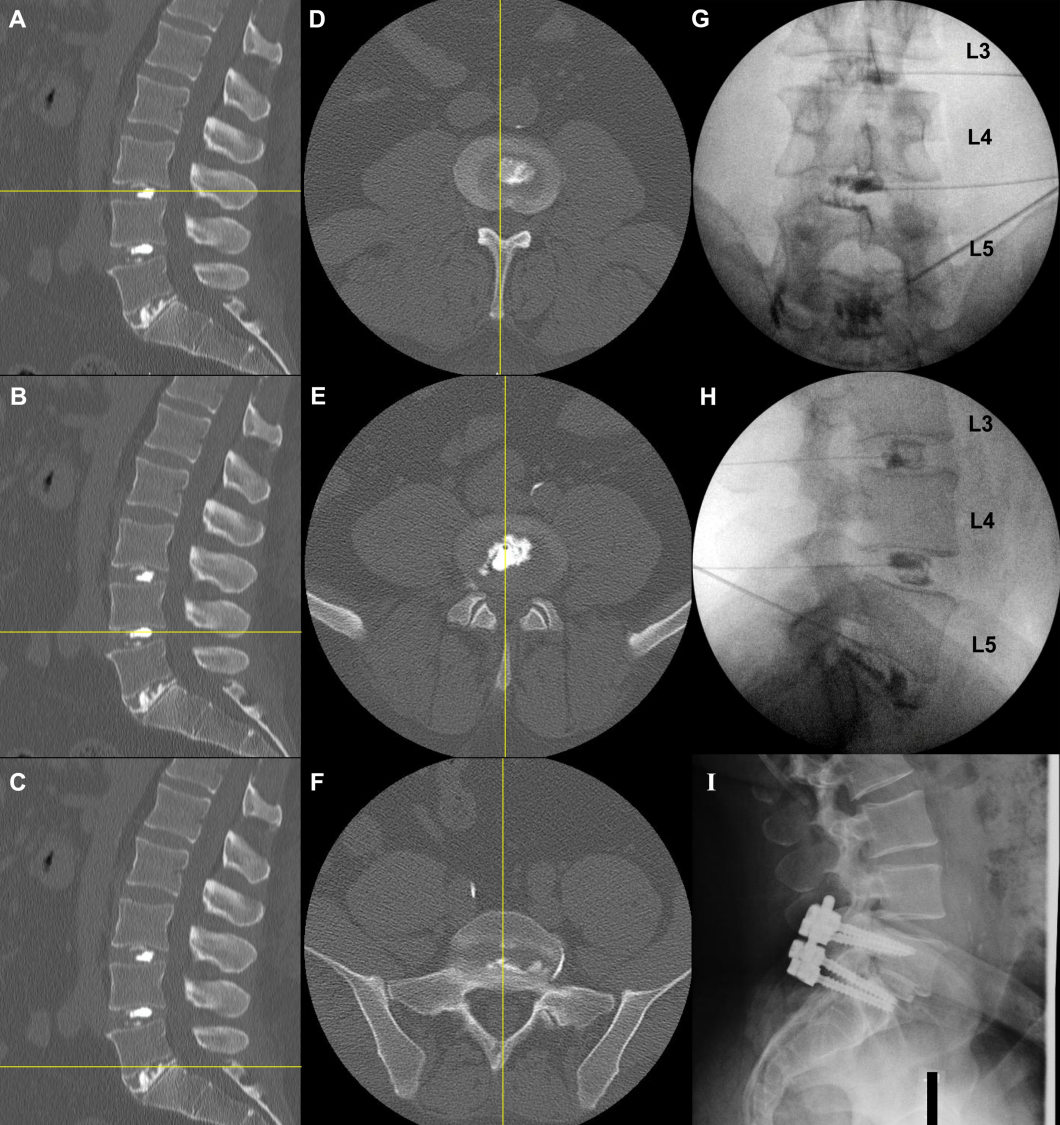
Post-discography CT (A-F), intra-discography (G, H), and post-surgical sagittal images (I) of a patient with single level of disc disruption. Sagittal (A-C) and axial (D-F) CT views show morphologically intact discs at L3-4 (A, D) and L4-5(B, E) with the radio-opaque dye contained within the nucleus pulposus, and structurally violateddisc at L5-S1 (C, F) with egressive dye into the spinal canal on the posterolateral aspect. Coronal(G) and sagittal (H) intra-discography radiographs illustrate structurally normal discs at L3-4 andL4-5; these discs showed no concordant pain. The L5-S1 disc had a damaged annulus thatpermitted contrast extravasation anteriorly, posteriorly, and laterally; it was painful uponprovocation. Interbody fusion with instrumentation at the L5-S1 level successfully andcompletely resolved the patient’s low back pain (I).

**Figure 4:**
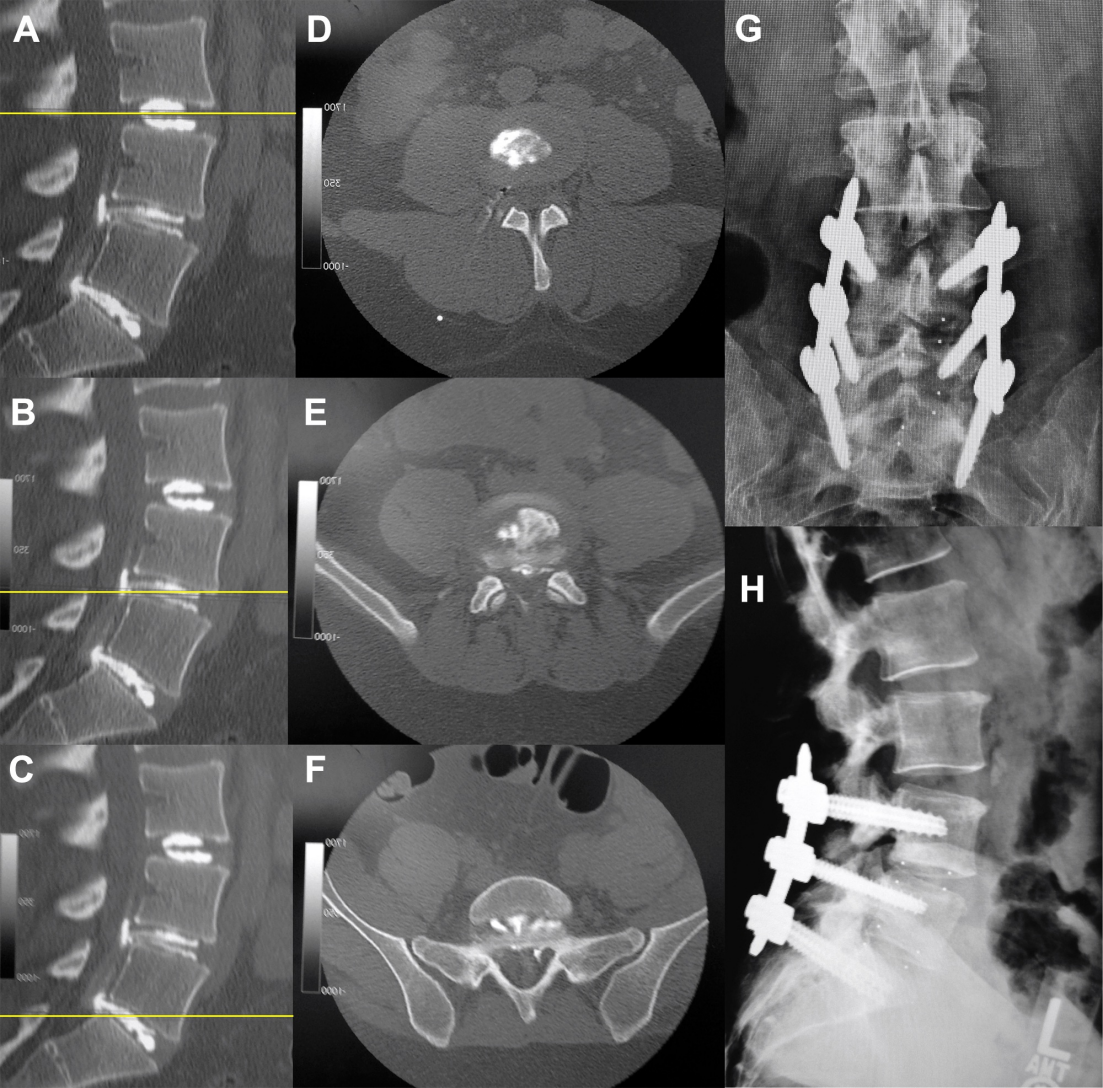
Post-discography CT (A-F) and post-surgical sagittal images (G, H) of a patient with two levels of disc disruption. Sagittal (A-C) and axial (D-F) CT views show morphologically intact discs at L3-4 (A, D) withradio-opaque dye contained within the nucleus pulposus, and structurally violated disc at L4-5(B, E) and L5-S1 (C, F) with dye egression into the spinal canal. Two-level interbody fusion withinstrumentation at L4-5 and L5-S1 successfully and completely resolved the patient’s low backpain (G, coronal view; H, sagittal view).

**Figure 5:**
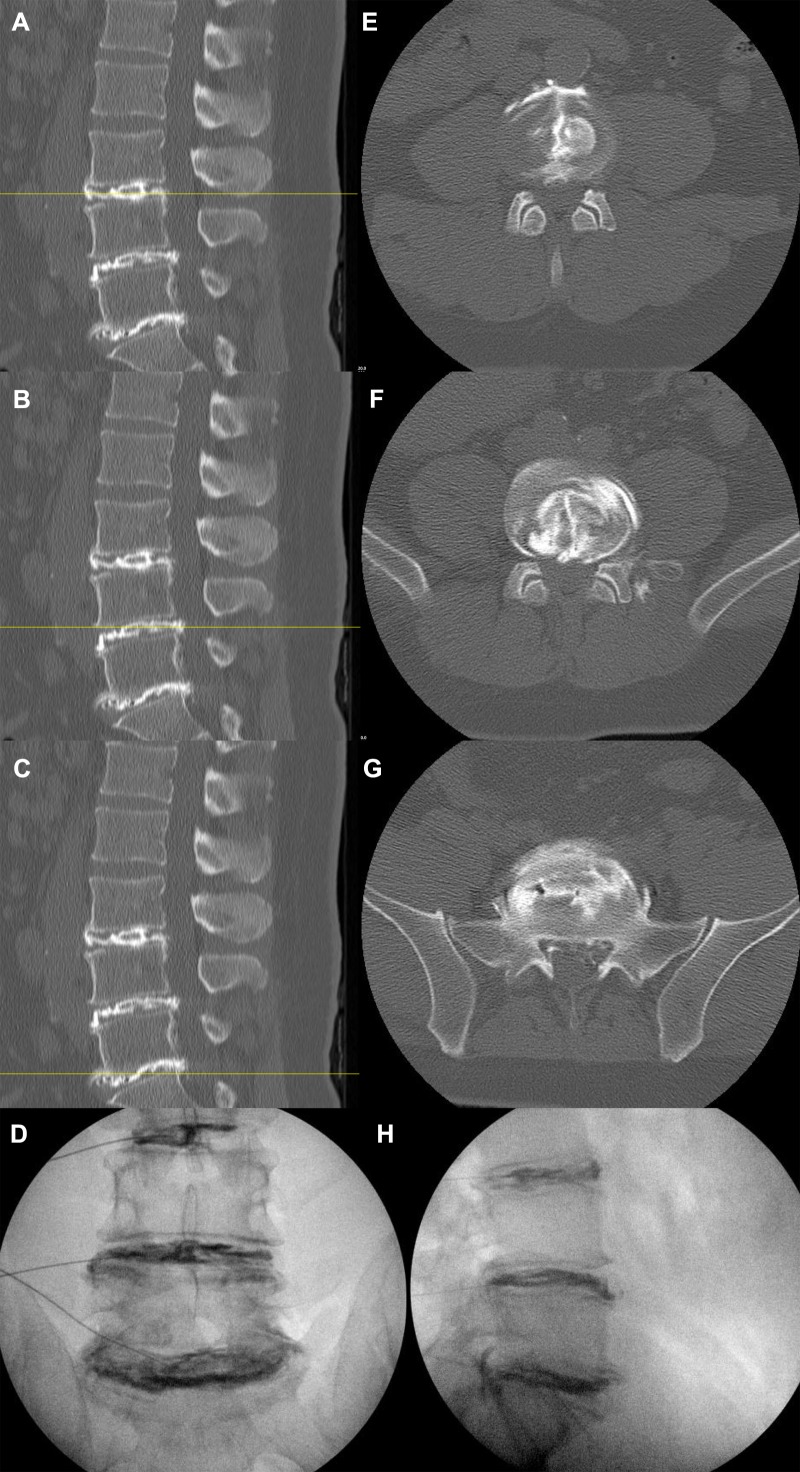
Post-discography CT (A-C, E-G) and intra-discography (D, H) images of a patient with extensive disc disruption at all three levels Sagittal (A-C) and axial (D-F) CT views show morphologically violated discs at L3-4 (A, D), L4-5 (B, E), and L5-S1 (C, F). Egressive radio-opaque dye into the spinal canal is seen at all 3 discs. Coronal (D) and sagittal (H) intra-discography radiographs illustrate structurally abnormal discs at all 3 levels, all of which showed concordant pain. Interbody fusion was not performed on this patient.

Intra-discography radiographs, post-discography CT, and post-surgical imaging of patients with one-level, two-level, and three-level disc disruptions are shown in Figures 3-5, respectively.

### Surgical outcomes

According to the selection criteria laid out in Figure 1, 18 patients, 10 (56%) men and 8 (44%) women, qualified for and underwent surgical intervention. The mean age was 48.8±12.3 (range, 34–82). Distribution of surgery levels was: L3-L4 (1), L4-L5 (6), L5-S1 (5), and contiguous two-level L4-S1 (6). Median follow-up time was 18 months (range, 12–78 months). Outcome results are summarized in Table 3.

**Table 3:** Outcome scores of visual analog scale (VAS), Short Form-36 (SF-36) Mental Component, SF-36 Physical Component, and Oswestry Disability Index (ODI), postoperative vs. preoperative

	Pre-Op	2 weeks	6 months	≥12 months
VAS	7.9±2.8	4.8±2.4	4.5±3.1	4.0±2.7
SF-36 Mental	39.8±9.3	43.6±8.4	53.6±9.7	52.0±8.5
SF-36 Physical	23.2±6.5	31.3±6.5	31.5±11.7	34.2±10.7
ODI	52.5±9.3	36.6±11.5	32.2±18.3	28.3±16.9

Notably, VAS and ODI scores significantly improved from preoperative baseline at 2 weeks, 6 months, and ≥12 months post-surgery (p<0.05). Both physical and mental components of SF-36 significantly improved from preoperative baseline at the same time points (p<0.05), except for mental component at 2 weeks and physical component at 6 months with discernable improvements that did not reach statistical significance. Overall, good to excellent outcomes (complete or near-complete symptomatic and functional recovery, with no or minimal analgesic use post-fusion) were achieved in 14 (78%) patients. The remaining 4 patients are accounted for in the discussion.

## Discussion

Provocative discography with subsequent CT provided basis for lumbar fusion in low-back-pain patients with an etiology of annular damage. In this study, concordant pain occurred significantly more often in discs with annular tears than those without. Non-painful discs occurred equally frequently in both groups, independent of annular status. These results mirror those previously published [9-11]. Contrast leakage from radial tears was clearly visualized on both intra- and post-discogram radiographs. The fissured discs were receptive to significantly higher amounts of injected dye than intact discs. This was likely due to extravasation of contrast dye through annular fibrosus tears. In the meantime, painful discs on discography also received significantly higher amounts of injected dye than painless discs. In most cases of positive concordant pain, posteriorly located, annular fibrosus tears were noted with egress of dye into the spinal canal. These findings suggest the egression of material (i.e., peptides, waste products) from the nucleus pulposus through the annular fibrosus may irritate the nerve roots and subsequently lead to back pain symptoms. This is consistent with the theory that extruded nuclear materials stimulate and sensitize nociceptors in the immediate vicinity via a cytokine-mediated inflammatory process [2,16-17].

Our data provided a rationale for fusion. Because we found a positive association between pain generation and annular disruption, we believed that arthrodesis of pathomorphological levels would be instrumental in pain relief. The use of post-discography CT was advantageous because of its superior resolution to visualize dye leakage compared with fluoroscopy alone [18]. Discs that were negative on both discography and imaging served as excellent controls to demarcate the range of pathology. We surgically treated those patients who were positive on both discography and subsequent CT at no more than two contiguous disc levels, with at least one level of negative control. This selection method yielded significant improvement of symptoms and function at almost all follow-up time points investigated. Immediate relief (at 2 weeks) and long-term amelioration (at or over 12 months) were both evident. More than three-quarters of the patients reported complete or almost complete resolution of symptoms up to years post-treatment, echoing results from earlier studies [7].

The subjective nature of reported pain is an important confounding factor that may create false positives in discography. Patients with psychometric abnormalities are known to have an exaggerated pain response upon stimulation [19-20]. Indeed, 3 of the 4 cases with unsatisfactory outcomes in this study, but not the 14 successful cases, were found to exhibit these characteristics. Of these 3 patients, one had persistent low back pain confounded by social situations and was referred to psychiatry. Two suffered depression and exhibited limited postoperative compliance. It is possible that these factors had led to falsely positive discography in the first place, resulting in poor surgical outcomes. We therefore believe that our primary hypothesis is not altered by these patients.

It is worth noting that provocative discography is not devoid of issues. It was recently found that discography was more likely than control to be associated with advancement of degenerative disc disease over a 10-year period [13]. The current study was not conducted over an interval long enough to address this issue. The site of new disc herniation correlated with the site of discography puncture. Meanwhile, new diagnostic tools have been attempted. Borthakur et al., reported significantly decreased T1rho MRI signals and significantly decreased discography opening pressures in painful discs compared with painless discs [21]. Zuo et al., demonstrated with magnetic resonance spectroscopy that elevated water/proteoglycan ratio correlated with symptomatic patients versus controls, and with positive discography versus negative discography [22]. In addition, mechanical microsensors [23], fiber optics microsensors [24], and proteoglycan osmodetectors [25] were employed to detect minute pressure or osmolarity changes in the IVD. However, these and other new advances have not been fully validated in a controlled clinical setting.

To date, there has not been a single, large-scaled, well-designed study that provides unequivocal evidence for or against the use of discography in conjunction with post-discography CT in providing scientific basis for surgical intervention. This is primarily due to the difficulty to control for subjective pain, the issue with sample size, the challenge of long-term follow-up, and perhaps most importantly the lack of a gold standard against which the current diagnostic protocol is compared. These limitations also apply to the current study. In addition, because no control was available for data analysis, it was impossible to conclude if surgical patients performed better than nonsurgical patients based on our criteria, or vice versa. Nonetheless, our data do suggest that discography with imaging is a useful tool that reliably generates significantly positive outcomes. While the ultimate etiology of discogenic pain remains elusive, it is important to factor into surgical decision making both the clinical and morphological manifestations; discography divulges the former and CT the latter. Although reliance on either one alone does not result in desirable outcome, a combination of both is one of the best currently available tools to identify painful degenerative discs.

## Conclusions

There was a significant association between concordant discogenic pain and annular tears as determined by discography and CT, respectively. Patients who received surgical management based on both positive discography and positive annular tears experienced symptomatic and functional improvements. This study suggests that, in the absence of a “gold standard”, discography in juxtaposition with CT remains a useful diagnostic tool in locating the generator of low back pain. Future research may focus on controlled prospective studies with larger sample size to examine the validity of this method.
